# Self-Esteem and Happiness as Predictors of School Teachers’ Health: The Mediating Role of Job Satisfaction

**DOI:** 10.3389/fpsyg.2018.00933

**Published:** 2018-07-17

**Authors:** Paula Benevene, Maya M. Ittan, Michela Cortini

**Affiliations:** ^1^Department of Human Sciences, LUMSA University, Rome, Italy; ^2^Department of Human and Social Sciences, Pontifical University of Saint Thomas Aquinas, Rome, Vatican City; ^3^Università degli Studi “G. d’Annunzio” Chieti-Pescara, Chieti, Italy

**Keywords:** subjective well-being, happiness, health, teachers, job satisfaction, self-esteem

## Abstract

**Background:** A wealth of cross-sectional studies show consistent positive relationships between teachers’ happiness and self-esteem on one hand, and health, on the other, which calls for additional research in order to disentangle cause and effect between the two, and to find potential mediators.

**Aims:** To investigate the mediating role played by job satisfaction between teachers’ happiness and self-esteem and their physical and mental health.

**Methods:** A questionnaire was administered, containing questions about participants’ background information and the following scales: the Job Satisfaction Survey, the Rosenberg Self-Esteem Scale, the Physical and Mental Health Scales (SF12), and the Ivens Scale in the Adapted Version for Teachers: School Children’s Happiness Inventory (SCHI). The participants were 300 primary and middle school teachers from the Indian State of Kerala.

**Results:** Job satisfaction fully mediates between both happiness and self-esteem, and health in teachers.

**Conclusion:** Work is a relevant domain to promote teachers’ happiness and self-esteem, and their health, through job satisfaction.

## Introduction

Happiness may be seen as the overall appreciation of one’s life-as-a-whole and several studies have proven that happiness promotes health; in fact, happiness has an effect on longevity, comparable to the effect of not smoking (Veenhoven, 2008). Unlikely the traditional clinical approach, the focus of happiness is on the individual’s own perspective, therefore happiness is commonly understood as a synonym of subjective well-being (SWB). SWB thus reflects the experience of a high level of positive affect, a low level of negative affect, and a high degree of satisfaction with one’s own life (Deci and Ryan, 2008). According to Diener et al. (1999) SWB is a multidimensional construct, involving both a cognitive component and an affective component. The relevance of observing narrower aspects of SWB lies in the fact that deepening the understanding of SWB in particular domains may suggest ways of improving the general level of individuals’ SWB, intervening on specific conditions that promote SWB in a specific sphere of activity. In such a sense, SWB can be observed either as a general satisfaction with one’s own life or as individual satisfaction in a specific domain, as the work domain.

Subjective well-being in the work experience can be therefore studied in relation to both the self or personality disposition and the affective components represented by self-esteem (Diener et al., 1999).

This latter is the affective response to one’s evaluation of self-worthiness, while job satisfaction is the psychological response of individuals to the value judgment of their job (Spector, 1997; Manuti and De Palma, 2017). Many studies have proven the relevant role played by self-esteem and job satisfaction in generating happiness (Bowling et al., 2010). In addition, self-esteem has proven to be strongly connected with job satisfaction (Schimmack and Diener, 2003).

Happiness or SWB is a key construct of the positive psychology, which aims at identifying the factors which promote and maximize individual wellness (Seligman and Csikszentmihalyi, 2000).

In particular, wellness and happiness of teachers may deserve scientific attention, being proved as able of impacting on students wellbeing and learning (Skaalvik and Skaalvik, 2011). Several studies during the last decades showed high rate of teachers’ attrition and ill-being, among Western and non-Western countries, in spite of the relevant differences among the educational systems and the relative paucity of studies carried out in countries outside European Union and United States (Hong, 2010).

Teachers’ happiness, along this vein, is a well-deserved matter of study, also because it refers on long-term states: in fact, several researches have proved that SWB have a degree of stability across years (Diener et al., 1997).

In terms of existing literature on teachers’ happiness or SWB, much attention has been devoted either to the issue of its relationship with burnout and work-related stress (Borrelli et al., 2014; Benevene and Fiorilli, 2015), or on the effects of stress and burnout on teachers’ health. On the contrary, to the authors’ knowledge, no previous studies have focused on the mediation role played by job satisfaction and self-esteem in the relationship between teacher’s happiness and their health.

Shedding a light on this mediation may deepen the understanding of the role played by dispositional and environment/contextual factors between happiness and health among teachers. It may also offer indications on how to promote teachers’ health in more efficient and effective ways, thus offering relevant information on which domains intervene to best promote SWB and health among teachers.

The present study is focused on the mediation role played by job satisfaction and self-esteem between happiness and general health among a group of schoolteachers from the Indian State of Kerala. We choosed Kerala because of the paucity of studies on teachers’ well-being in India and, more in general, in Asian and non-Western countries. On the other hand, findings emerged till now confirmed the validity of the conceptualization of happiness, in spite of the differences due to the cultures and level of economic development (Diener and Tov, 2012). Findings from this study, in fact, might offer indication on future research carried out in other cultural contexts.

In this respect, it has to be underlined that SWB has emerged as an effective instrument to (a) overcome the limitations of the knowledge based on psychological studies, referred mainly to negative and pathological states; (b) to set up effective policies dealing with public health (Ryan and Deci, 2001).

Therefore the following hypothesis were tested:

Hypothesis 1: Happiness positively affects health of schoolteachers.

The first hypothesis, actually, lies on a long tradition within psychology literature (see, for example, Lyubomirsky et al., 2005), with recent findings concerning work and organizational psychology, where, with different methodologies and tools, ranging form very complex data collection based, for example on the Minnesota Satisfaction Questionnaire (Weiss et al., 1967) to single-item scale studies (among others, see Cortini, 2016), happiness seems to positively affect workers’ health.

Hypothesis 2: Self-esteem positively influences health of schoolteachers.

The relationship between self-esteem and health has actually been widely studied, especially within adolescence literature (see, for example, Trzesniewski et al., 2006).

Hypothesis 3: Job satisfaction mediates the relationship between happiness and health for schoolteachers.

Happiness and life satisfaction have been already found to be related to health, especially within the positive psychology literature (see, for example, Youssef and Luthans, 2007) that we explicitly followed. Here our concern is with a specific population, Indian teachers, up to now under-studied.

Hypothesis 4: Job satisfaction mediates the relationship between self-esteem and health for schoolteachers.

Finally, following the results of an interesting review on stress prevention (Lambert and Lambert, 2001), we tested a model of mediation where job satisfaction impacts on the relationship between self-esteem and health for schoolteachers.

## Materials and Methods

### Participants

A total of 300 teachers were randomly selected to freely participate in the study; 92% (*N* = 276) were female. All participants were full-time school members.

A stratified proportionate random sampling model was adopted. Three non-overlapping strata were considered: type of school (state and private schools), level of school (primary, middle, and high schools), and the teaching experience (0–5 years, 5–10 years, more than 10 years).

### Instrument and Measures

An anonymous questionnaire was administered, following the principal’s permission of the 10 schools involved, ranging from primary to high school. The questionnaire contained a cover letter, explaining the purpose of the study and the essence of each scale; all participants were assured about the confidentiality of their responses. Further information was given prior the administration of the questionnaire, to present and fully explain the aim of the study; each participant gave informed consent prior to being included in the study.

The questionnaire was administered individually. The first part of the questionnaire contained questions about participant’s socio-demographic information that is: sex, age, years of teaching, type of school where participant worked; the second part contained the following scales: the Job Satisfaction Survey (JSS) (Spector, 1985), the Rosenberg Self-Esteem Scale (RSES) (Rosenberg, 1965), the Physical and Mental Health Scales (SF12) (Ware et al., 1996), and the Ivens Scale in the adapted version for teachers: School Children’s Happiness Inventory (SCHI) (Ivens, 2007).

**The JSS** measures respondent’s satisfaction about his/her job situation. It comprises 36 items, divided among nine facets or sub-scales: pay, promotion, supervision, fringe benefits, contingent rewards, operating procedures, co-workers, nature of work, and communication. Items were rated using a six-point Likert scale, ranging from: 1 = “I disagree very strongly” to 6 = “I agree very strongly.” Total job satisfaction is expressed as a total score ranging from 36 (low job satisfaction) to 216 (high job satisfaction).

**The RSES** comprises 10 items and is commonly used as an empirical measure of a person’s overall self-esteem. Items were rated using a 4-point Likert scale, ranging from 1 = I strongly disagree, to 4 = I strongly agree. The higher the score, the higher the self-esteem. Scores between 15 and 25 are within normal range; scores below 15 suggest low self-esteem.

#### Physical and Mental Health Scales SF12

The SF12 is a multipurpose short form survey with 12 questions, all selected from the SF-36 Health Survey (Ware et al., 1996). The scale contains several sub-scales: one sub-scale using a 3-point Likert scale to assess limitations in physical activity and physical role functioning; two sub-scales using a 5-point Likert scale assessing, respectively, pain and overall health; a sub scale using 6-point Likert scale, assessesing mental health, vitality, and social functioning; the scale contains also questions with yes/no answers, to assess limitations in role functioning as a result of physical and emotional health. The Physical and the Mental Health Composite Scores (PCS and MCS) are computed using the scores of the 12 questions. The possible score range from 0 to 100, where higher scores represent better health.

All instruments showed satisfactory reliability, with an Alpha of Cronbach ranging from 0.63 to 0.89.

Local academic rules do not require ethical approval for studies on non-clinical populations. However, the study was approved by the schools’ management, following the ethical code of the Italian Association of Psychology.

### Data Analysis

Preliminary analyses were performed to ensure there was no violation of the assumption of normality, linearity, and multicollinearity in the dataset, resulting in good distributive qualities.

The associations between the study variables were analyzed by a multiple linear regression, and a significant regression equation was found [*F*(2.297) = 56.08; *p* < 0.001], with an *R*^2^ = 0.46, showing that happiness and self-esteem were significant predictors of schoolteachers’ health, with, respectively, the following β values: 0.359^∗∗∗^ and 0.469^∗∗∗^.

A mediation analysis was then performed, using the SPSS macro PROCESS, to verify the indirect effects of the predictor on the dependent variable. Bootstrap confidence intervals were used in consideration to the limited sample size.

All variables were mean centered before running the analysis, to minimize the risk of multicollinearity.

## Results

**FIGURE 1:**
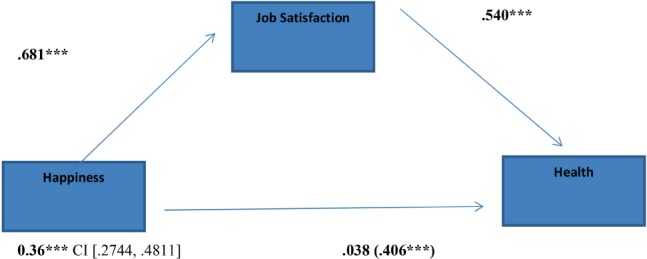
Indirect effect of happiness on health through job satisfaction; ^∗^*p* < 0.05; ^∗∗^*p* < 0.01; ^∗∗∗^*p* < 0.001.

Results show that that job satisfaction fully mediates the relation between happiness and health (**Figure 1**) [indirect effect = 0.368, *SE* = 0.052, 95% CI (0.2744, 0.4811)]; in fact, zero is not in the 95% confidence interval, and the indirect effect is significantly different from zero at *p* < 0.05.

The mediator could account for the majority of the effect; PM = 0.90; Sobel *z* = 7,55 [*p* < 0.001; *k*^2^ = 0.29, CI (0.2285, 0.3578)].

**FIGURE 2:**
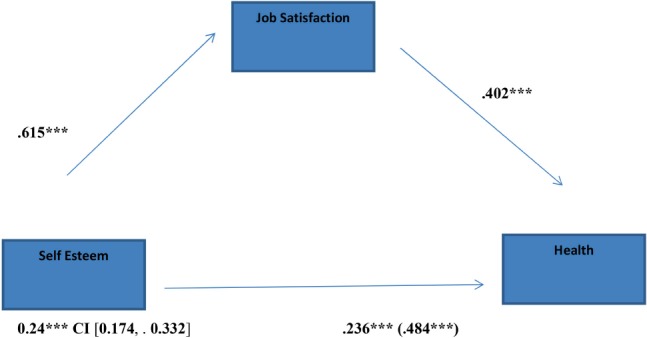
Indirect effect of self-esteem on health through job satisfaction; ^∗^*p* < 0.05; ^∗∗^*p* < 0.01; ^∗∗∗^*p* < 0.001.

Results show also that job satisfaction significantly mediated the relation between self-esteem and health (**Figure 2**) [indirect effect = 0.247, *SE* = 0.039, 95% CI (0.174, 0.332)]: zero is not in the 95% confidence interval, therefore it can be concluded that the indirect effect is significantly different from zero at *p* < 0.05.

The mediator could account for almost half of the effect; PM = 0.51; Sobel *z* = 6,22 [*p* < 0.001; *k*^2^ = 0.23, CI (0.1789, 0.3055)].

## Discussion

To the authors’ knowledge, the mediation role played by job satisfaction has not been addressed before in conjunction with teachers’ happiness and their health. Results show that job satisfaction fully mediates the relationship between teachers’ happiness and health, as well as the relationship between their self-esteem and health. These findings confirm the relationship between happiness and health, as well as the relevant role played by job satisfaction and self-esteem in this regard. However, from no previous study emerged that self-esteem – that is a dispositional trait – emerged as mediated by job satisfaction – that is a focal domain satisfaction and the cognitive components of happiness – in determining teachers’ health.

Findings suggest that dispositional traits of SWB have an impact on teachers’ health but, at the same time, cognitive, and environment factors play a role not only on health, but also on dispositional traits which, in turn, impact on health.

These results suggest the relevance of developing policies aimed at promoting job satisfaction among schoolteachers, intervening on their work condition, in order to promote their health. In addition, our results call for a deepened understanding of schoolteachers’ job unsatisfaction, which could be investigated with qualitative research.

More in general, evidences emerged from this study confirmed the consequences of happiness on health, in spite of the differences due to the cultures and level of economic development (Diener and Tov, 2012).

On the one hand, the study addressed the paucity of studies on teachers’ well-being in Asian and non-Western countries; on the other, our findings confirm the relationship between happiness and health, as well as the relevant role played by job satisfaction and self-esteem on happiness. There is strong evidence in literature, to whom our results make an echo, that schoolteachers are suffering from psychological, mental, and physical problems, among both Western and non-Western countries, despite the differences between the educational systems (Skaalvik and Skaalvik, 2011).

Though promising, our findings cannot be generalized, since the group reached is not a statistically representative sample and is limited to India. In the future, it would be interesting to administrate the questionnaire reaching a statistically representative sample and comparing the findings at a cross-cultural level. Further analysis could investigate the relationships in question with a longitudinal perspective. This could correct potential bias due to the cross-sectional design, and to shine light on the role played by aging, which has recently been found to be a very important variable in the teaching domain (Avanzi et al., 2012; Converso et al., 2018).

In addition, it seems to us mandatory to study the role played by organizational support that can have a severe impact on job satisfaction and health, resulting in an additional mediator, as we have shown elsewhere (Cortini et al., 2016).

Last but not least, it would be interesting to investigate the role of job crafting in the variables relationship under analysis, following a very promising line of research in W/O psychology (see, for example, the recent paper by Ingusci et al., 2016), along with the role played by new technologies (Manuti and De Palma, 2017).

## Author Contributions

MI administered the questionnaire and prepared the data set for the analysis. MC analyzed the data and wrote the Sections “Materials and Materials” and “Results.” PB was in charge of the research design and wrote the Sections “Introduction” and “Conclusion.”

## Conflict of Interest Statement

The authors declare that the research was conducted in the absence of any commercial or financial relationships that could be construed as a potential conflict of interest.
